# Metabolic Network Topology of Alzheimer’s Disease and Dementia with Lewy Bodies Generated Using Fluorodeoxyglucose Positron Emission Tomography

**DOI:** 10.3233/JAD-190843

**Published:** 2020-01-07

**Authors:** Masamichi Imai, Mika Tanaka, Muneyuki Sakata, Kei Wagatsuma, Tetsuro Tago, Jun Toyohara, Renpei Sengoku, Yuji Nishina, Kazutomi Kanemaru, Kenji Ishibashi, Shigeo Murayama, Kenji Ishii

**Affiliations:** a Team for Neuroimaging Research, Tokyo Metropolitan Institute of Gerontology, Tokyo, Japan; b Toranomon Hospital, Tokyo, Japan; c Department of Neurology, Tokyo Metropolitan Geriatric Hosptal and Institute of Gerontology, Tokyo, Japan

**Keywords:** Alzheimer’s disease, dementia with Lewy bodies, fluorodeoxyglucose, graph theory, network analysis, neuroimaging biomarkers, positron emission tomography

## Abstract

**Background:**

Alzheimer’s disease (AD) and dementia with Lewy bodies (DLB) are often misdiagnosed with each other because of similar symptoms including progressive memory loss. The metabolic network topology that describes inter-regional metabolic connections can be generated using fluorodeoxyglucose positron emission tomography (FDG-PET) data with the graph-theoretical method. We hypothesized that different metabolic connectivity underlies the symptoms of AD patients, DLB patients, and cognitively normal (CN) individuals.

**Objective:**

This study aimed to generate metabolic connectivity using FDG-PET data and assess the network topology to differentiate AD patients, DLB patients, and CN individuals.

**Methods:**

This study included 45 AD patients, 18 DLB patients, and 142 CN controls. We analyzed FDG-PET data using the graph-theoretical method and generated the network topology in AD patients, DLB patients, and CN individuals. We statistically assessed the topology with global and nodal parameters.

**Results:**

The whole metabolic network was preserved in CN; however, diffusely decreased connection was found in AD and partially but more deeply decreased connection was observed in DLB. The metabolic topology revealed that the right posterior cingulate and the left transverse temporal gyrus were significantly different between AD and DLB.

**Conclusion:**

The present findings indicate that metabolic connectivity decreased in both AD and DLB, compared with CN. DLB was characterized restricted but deeper stereotyped network disruption compared with AD. The right posterior cingulate and the left transverse temporal gyrus are significant regions in the metabolic connectivity for differentiating AD from DLB.

## INTRODUCTION

Alzheimer’s disease (AD) and dementia with Lewy bodies (DLB) are the two primary forms of neurodegenerative dementia [1]. They are different in terms of clinical course, therapeutic management, and prognosis, as well as clinical diagnosis criteria; however, AD and DLB are often misdiagnosed with each other because both are characterized by memory loss and cognitive impairments in perception, spatial function, and constructive abilities [2–4].

Fluorodeoxyglucose positron emission tomography (FDG-PET) is commonly used for evaluating brain function. Glucose metabolism decreases in dementia including AD and DLB, compared with cognitively normal (CN) individuals. For differentiating DLB from AD, we can focus on hypometabolism in the visual field [5] and on relatively preserved metabolism in the posterior cingulate cortex (cingulate island sign [6]); however, we have to consider metabolic pattern overlaps in quite a few cortices in AD and DLB [7].

Brain networks have recently become a hot topic in the neuroscience field. Brain networks with the graph-theoretical method provide a mathematical model for quantifying structural and functional connectivity. They are studied generally using blood oxygen level-dependent (BOLD) signals that are synchronized between brain regions and functional magnetic resonance imaging (MRI) [8], which is generally conducted concomitantly in a certain task. Brain stimulation induced by the task increases regional blood supply [9]. The correlations between different activated brain regions can be visualized as simultaneous changes of blood supply (BOLD signals) with functional MRI. In addition, blood supply delivering oxygen and glucose metabolism are strongly correlated [10], and resting-state oxygen consumption and glucose utilization are also correlated [11]. Accordingly, we hypothesized that metabolic connectivity can be generated using FDG-PET data, instead of functional MRI data, with the graph-theoretical method to visualize inter-regional metabolic activation.

It has been demonstrated that the functional connectivity obtained by functional MRI shows a specific pattern for AD. One of the most important connections impaired in AD is the default mode network (DMN) involving in episodic memory processing [12–15]. In addition, dysfunction of the DMN in mild cognitive impairment (MCI) can be used to predict the conversion from MCI to AD [16].

However, the functional connectivity in DLB obtained by functional MRI is inconsistent. For instance, Galvin et al. found significant differences in the functional connection of the precuneus to the primary visual cortex not only between DLB and CN, but also between DLB and AD [17]. However, Kenny et al. found no significant differences in this connection among DLB, AD, and CN [18].

In this study, we hypothesized that the metabolic connectivity obtained by FDG-PET differs among AD, DLB, and CN and could be used to differentiate among them. Therefore, this study aimed to assess the metabolic connectivity using FDG-PET with the graph-theoretical method to differentiate among AD, DLB, and CN.

## MATERIALS AND METHODS

### Participants

This study included 45 AD patients (30 females, 69±11 years; 15 males, 70±10 years), 18 DLB patients (6 females, 81±4 years; 12 males, 75±8 years), and 142 CN controls (127 females, 67±5 years; 15 males, 66±5 years) (Table 1). Participants were recruited from those who underwent both FDG-PET and MRI in our hospital from June 2000 to May 2014. Dementia was diagnosed according to the criteria of the fourth edition of the Diagnostic and Statistical Manual of Mental Disorders (DSM-IV). All patients with AD were classified as having probable AD with a high level of biomarker evidence based on “Recommendations from the National Institute on Aging-Alzheimer’s Association workgroups on diagnostic guidelines for Alzheimer’s disease” published in 2011 (NIA-AA 2011) [19]. Amyloid-β biomarkers were assessed using [^11^C]PiB-PET. DLB was diagnosed according to the criteria of the fourth report of the DLB Consortium [20]. CN controls had no cognitive impairment and were not taking any medications targeting at the central nervous system. Patients with notable organic brain lesions were excluded from this study. All participants provided written informed consent. This study was approved by the Institutional Ethics Committee of Tokyo Metropolitan Institute of Gerontology.

**Table 1:** Characteristics of participants

Group	AD patients	DLB patients	CN individuals
Number	45	18	142
Age (y)	69±11	77±7	67±5
Female	67% (*n* = 30)	33% (*n* = 6)	89% (*n* = 127)
MMSE	21±7	23±4	29±1

All AD patients were diagnosed as having probable AD with high levels of biomarker probability according to NIA-AA 2011. All DLB patients were diagnosed using the fourth consensus report (the newest version) of the DLB Consortium. AD, Alzheimer’s disease; DLB, dementia with Lewy bodies; CN, cognitive normal; MMSE, Mini-Mental State Exam.

### FDG-PET imaging

PET studies were performed using the Headtome-V/SET 2400 W Scanner (Shimadzu, Kyoto, Japan). All patients fasted at least 5 h before the PET study. They were kept still on the bed and were then prepared for intravenous catheter insertion. A bolus of 150–185 MBq [^18^F]-FDG was administered in participants with a target serum glucose level of less than 140 mg/dL. Attenuation was corrected by a transmission scan with ^68^Ga/^68^Ge rotating source before the emission scan. A 12-min emission scan in a 3D acquisition mode was started at 45 min after the injection. PET images were reconstructed using a filtered back projection method and Butterworth filter (cutoff frequency, 1.25 cycle/cm; order, 2).

### Data processing

All of the PET data were preprocessed using Statistical Parametric Mapping 8 (SPM8) software (Welcome Department of Imaging Neuroscience, University College, London, UK) implemented in the MatLab (Mathworks Inc, MA, USA). All of the PET images were spatially normalized to the Montreal Neurological Institute (MNI) stereotactic space and smoothed by convolution with an isotropic Gaussian filter with 16 mm full width at half maximum (FWHM). Standard uptake value (SUV) was calculated for each voxel, and the SUV ratio (SUVR) images were then generated compared with the mean SUV of the cerebellar cortex.

### Graph-theoretical descriptive measures

To define the nodes in the graph-theoretical method, we segmented the SUVR plot to different brain regions according to an anatomical atlas of FreeSurfer version 5.1 in order to project the regions equivalent for the nodes on the spatial coordinates. The list of the regions for further analysis is described in Table 2. Subsequently, the Pearson correlation between the values of all pairs of the brain regions was calculated. The correlation denotes a connectivity matrix that represents the strength of the connection between a pair of nodes. All of the graph measures were introduced using optimized algorithms based on linear algebra to generate network construction. Graph-theoretical measures, which are used to assess the topology of the global network and its regions, were calculated in each group to adopt a method involved in Brain Connectivity Toolbox (http://www.brain-connectivity-toolbox.net/). The calculation was performed using BRAPH software based on MatLab platform [21]. The software can be used to assess the correlation in all pairs of regions according to the anatomical atlas, compute the network topology, and calculate the graph measures to describe the character of the topology. We used the BrainNet Viewer for network visualization [22].

**Table 2:** Brain regions involved in the graph-theoretical method

No	Regions	Montreal neurological institute coordinates			L/R	label
		x	y	z
1	superior frontal	–12.6	22.9	42.4	left	lSF
2	frontal pole	–8.6	61.7	–8.7	left	lFP
3	rostral middle frontal	–31.3	41.2	16.5	left	lRMF
4	caudal middle frontal	–34.6	10.2	42.8	left	lCMF
5	pars orbitalis	–41	38.8	–11.1	left	lPOB
6	lateral orbitofrontal	–24	28.6	–14.4	left	lLOF
7	pars triangularis	–42.4	30.6	2.3	left	lPT
8	pars opercularis	–44.6	14.6	13.1	left	lPOP
9	medial orbitofrontal	–8	34.9	–14.9	left	lMOF
10	rostral anterior cingulate	–6.8	33.9	1.6	left	lRAC
11	caudal anterior cingulate	–6.6	18	26.1	left	lCAC
12	insula	–34.2	–4.3	2.2	left	lINS
13	precentral	–37.8	–10.7	42.1	left	lPRC
14	postcentral	–42.3	–23.8	43.6	left	lPOC
15	supramarginal	–50.4	–38.8	31	left	lSUPRA
16	superior parietal	–22.8	–60.9	46.3	left	lSP
17	inferior parietal	–40	–66.4	27.3	left	lIP
18	paracentral	–10	–28.7	56.1	left	lPARAC
19	posterior cingulate	–7.3	–17.4	35.7	left	lPCG
20	isthmus cingulate	–8.9	–45.4	17.6	left	lIST
21	precuneus	–11.6	–57.5	36.7	left	lPREC
22	cuneus	–8.7	–79.6	18	left	lCUN
23	pericalcarine	–13.9	–80.6	6	left	lPERI
24	lingual	–16.5	–66.8	–4.3	left	lLIN
25	lateral occipital	–29.7	–86.9	–1	left	lLO
26	transverse temporal	–44	–24.2	6	left	lTRANS
27	banks superior temporal	–52.7	–44.5	4.6	left	lBKS
28	superior temporal	–52.1	–17.8	–4.4	left	lST
29	middle temporal	–55.6	–31.1	–12.9	left	lMT
30	inferior temporal	–48.9	–34.4	–22.2	left	lIT
31	temporal pole	–32.8	8.4	–34.8	left	lTP
32	entorhinal	–25.8	–7.6	–31.6	left	lENT
33	parahippocampal	–24.7	–31.2	–17.4	left	lPHIP
34	fusiform	–35.7	–43.3	–19.7	left	lFUS
35	superior frontal	13.4	24.7	42	right	rSF
36	frontal pole	10.3	61.1	–10	right	rFP
37	caudal anterior cingulate	7.3	18.7	26.3	right	rCAC
38	caudal middle frontal	34.9	11.8	43	right	rCMF
39	pars orbitalis	42.1	39.2	–10	right	rPOB
40	lateral orbitofrontal	23.6	28.5	–15.2	right	rLOF
41	pars triangularis	45	29.7	4.5	right	rPT
42	pars opercularis	44.9	14.4	14.2	right	rPOP
43	medial orbitofrontal	8.8	35.7	–14.8	right	rMOF
44	rostral middle frontal	32.3	40.9	17.3	right	rRMF
45	rostral anterior cingulate	8	33.5	2.1	right	rRAC
46	insula	35.1	–3.9	2.4	right	rINS
47	precentral	36.8	–9.9	43.5	right	rPRC
48	postcentral	41.6	–22.4	43.8	right	rPOC
49	supramarginal	50.6	–33.3	30.7	right	rSUPRA
50	superior parietal	22.6	–59.5	48.1	right	rSP
51	inferior parietal	42.8	–60.9	28.1	right	rIP
52	paracentral	9.9	–27.4	55.6	right	rPARAC
53	posterior cingulate	7.6	–17.1	36.2	right	rPCG
54	isthmus cingulate	9.8	–44.8	16.9	right	rIST
55	precuneus	11.7	–56.5	37.7	right	rPREC
56	cuneus	8.7	–80.1	19	right	rCUN
57	pericalcarine	14	–79.7	6.7	right	rPERI
58	lingual	16.8	–66.3	–3.6	right	rLIN
59	lateral occipital	30.3	–86.3	0.5	right	rLO
60	transverse temporal	44.8	–22.4	6.5	right	rTRANS
61	banks superior temporal	51.9	–40.6	5.6	right	rBKS
62	superior temporal	53	–14	–5.5	right	rST
63	middle temporal	55.9	–29.5	–12.9	right	rMT
64	inferior temporal	49.3	–31.7	–23	right	rIT
65	temporal pole	34	8.4	–33.1	right	rTP
66	entorhinal	26.2	–6.8	–31.9	right	rENT
67	parahippocampal	26.1	–31.3	–16.2	right	rPHIP
68	fusiform	35.9	–43	–19.2	right	rFUS

### Network analysis

To assess the global network topology in AD, DLB, and CN, we calculated the following global parameters: 1) average strength, the average nodal strength calculated by the sum of the weights of all connections of the node; 2) average eccentricity, the average nodal maximal shortest path length between a node and any other node; 3) average characteristic path length, the average of the shortest path lengths between one node and all nodes; 4) average global efficiency, the average inverse shortest path length; 5) average local efficiency, the average inverse shortest path length between one node and node’s neighborhood; 6) average clustering coefficient, the average nodal fraction of the degree within its neighborhood over the number of the connections that possibly exist between them; 7) transitivity, the fraction of the numbers of the triangles over the total number of the triplets; and 8) modularity, a statistic that quantifies the degree to which the topology can be divided into subnetworks. To assess the regional network, we calculated the following nodal parameters for each node: 1) nodal degree, total number of edges connected to the node; 2) nodal strength, the sum of the weights of all connections of the node; 3) triangles, the number of the neighboring nodes that link to each other, resulting in triangle form between a node and its neighbors; 4) nodal eccentricity, the maximal shortest path length between the node and any other node; 5) nodal path length, the shortest path lengths between the node and all the other nodes; 6) nodal clustering coefficient, fraction of the degree within its neighborhood over the number of the connections that possibly exist between them; 7) global efficiency of the node, average of the inverse shortest path length from a node to all other nodes; and 8) closeness centrality, inverse of the path length of the node.

A non-parametric permutation test was performed to assess the global and nodal parameters in AD, DLB, and CN. A *p*-value of <0.05 after controlling for the family-wise error rate was considered significant with a two-tailed test of the null hypothesis.

## RESULTS

**Fig.1:**
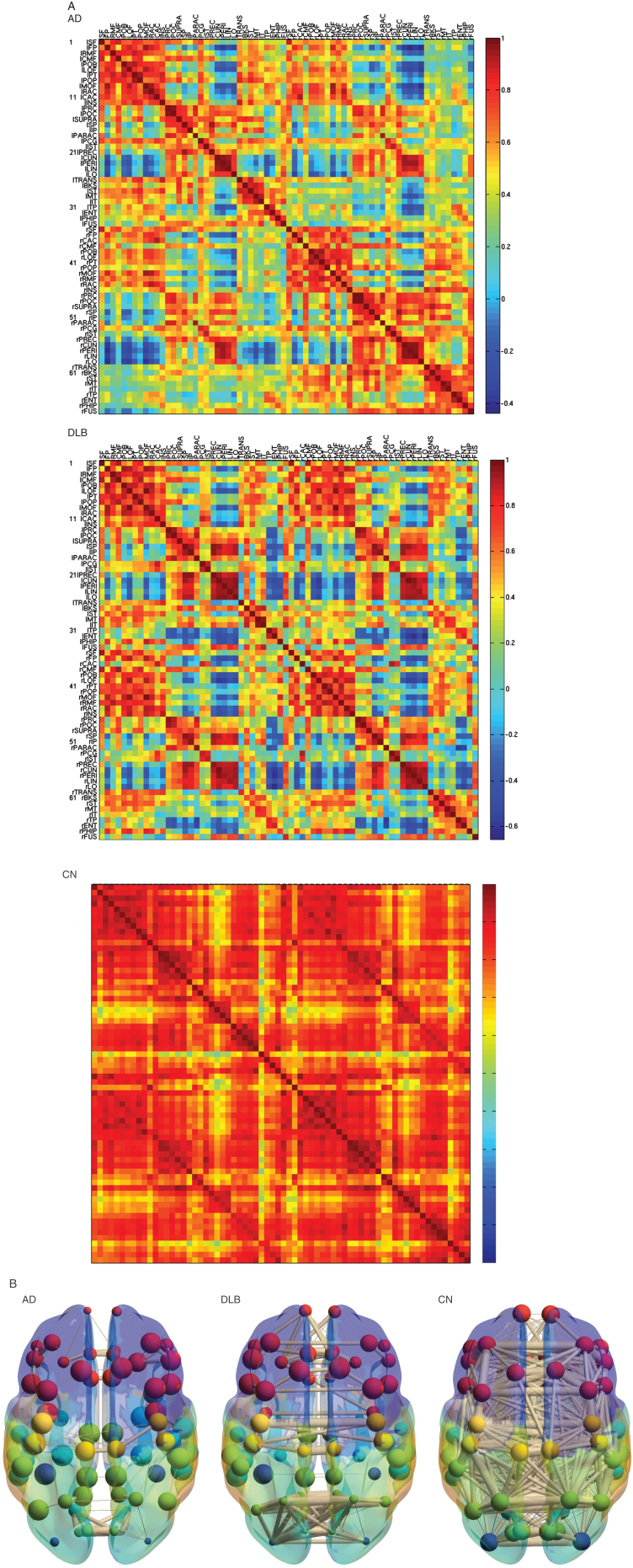
Metabolic connectivity matrix and anatomical localizations. A) Metabolic connectivity matrix. The cell color in the correlation matrix indicates the magnitude of the correlation, and the color is arranged in gradation from red to blue in accordance with the magnitude of correlation from positive to negative. Note that the lower limit of the correlation range is different in each matrix (See a color navigation side bar). Some of the correlations decreased more severely in DLB than in AD. In addition, other correlations in DLB are relatively preserved. The texture of the matrix in DLB looks “patchy” compared with that in AD. AD, Alzheimer’s disease; DLB, dementia with Lewy bodies; CN, cognitive normal. B) The 3D schematic figures representing metabolic connectivity. The metabolic connections are overlaid on an anatomical atlas using nodes and edges. These figures are used to display an outline of the whole connectivity.

The metabolic correlation matrix in CN was highly homogeneous in the whole brain; in contrast, the correlation matrices in AD and DLB were heterogeneous including lower correlation (Fig. 1). Some of the correlations decreased severely in DLB compared with AD; however, other correlations in DLB were partially preserved. The 3D schematic figures clearly visualized the difference of the network topology between AD and DLB.

**Fig.2:**
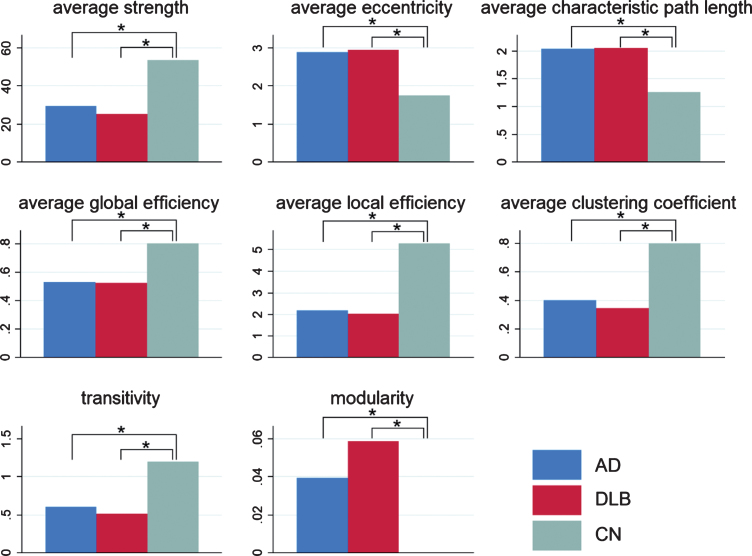
Global parameters. Global parameters, including average strength, average eccentricity, average characteristic path length, average global efficiency, average local efficiency, average clustering coefficient, transitivity, and modularity, are displayed in the bar chart with blue bars for AD, red bars for DLB, and green bars for CN. In all the parameters, significant differences were found in AD versus CN and DLB versus CN, but no significant difference was found between AD and DLB. *p < 0.05

In the global parameters (Fig. 2), average strength, global efficiency, local efficiency, clustering coefficient, and transitivity were significantly lower in AD than in CN. Average eccentricity, average characteristic path length, and modularity were significantly higher in AD than in CN. Similar results were obtained when DLB was compared with CN. Average strength, global efficiency, local efficiency, clustering coefficient, and transitivity were lower, and average eccentricity, average characteristic path length, and modularity were higher in DLB than in CN. However, no significant difference in the global parameters was found between AD and DLB.

**Fig.3:**
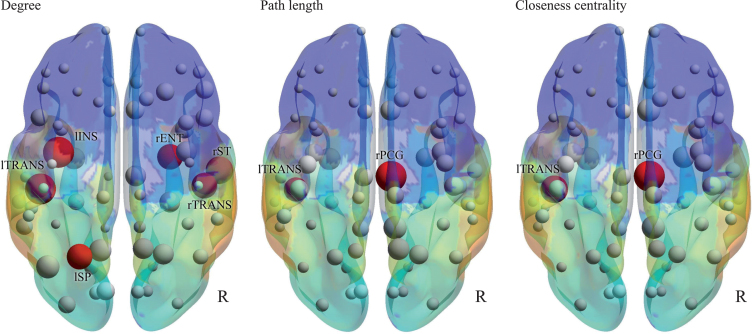
Nodal parameters. Degree, path length, and closeness centrality are shown by 3D schematic figures. Red nodes indicate significant differences between AD and DLB, corresponding to Table 3.

In the nodal parameters (Fig. 3 and Table 3), significant differences in nodes were found between AD and DLB. The most remarkable node was the right posterior cingulate, which had lower strength, lower triangles, higher path length, lower global efficiency, lower clustering coefficient, and lower closeness centrality in DLB than in AD. The second most remarkable node was the left transverse temporal gyrus, which had a lower degree, higher path length, and lower closeness centrality in DLB than in AD.

**Table 3:** Nodal parameters with significant differences between AD and DLB

Brain regions	Measures	AD	DLB	Difference	*p*
right posterior cingulate	strength	38.02	15.69	–22.33	<0.01
right posterior cingulate	triangles	1049.5	372.9	–676.6	0.03
right posterior cingulate	path length	1.8538	3.2901	1.4363	<0.01
right posterior cingulate	global efficiency of the nodes	0.5688	0.3494	–0.2194	<0.01
right posterior cingulate	clustering nodes	0.4747	0.2256	–0.2491	0.02
right posterior cingulate	closeness centrality	0.5394	0.3039	–0.2355	<0.01
left transverse temporal	degree	67	50	–17	<0.01
left transverse temporal	path length	2.0527	2.7779	0.7252	0.04
left transverse temporal	closeness centrality	0.4872	0.3600	–0.1272	0.02
left insula	degree	67	51	–16	<0.01
left superior parietal	degree	58	41	–17	<0.01
right transverse temporal	degree	67	52	–15	<0.01
right superior temporal	degree	67	52	–15	<0.01
right entorhinal	degree	67	40	–27	<0.01

## DISCUSSION

We generated the metabolic connectivity using FDG-PET with the graph-theoretical method. The metabolic connections decreased in AD and DLB compared with CN. The patterns of the decreased metabolic connections were different between AD and DLB, as shown by the difference in the nodal parameters of the specific nodes including the right posterior cingulate and the left transverse temporal gyrus.

In this study, patients clinically diagnosed with AD underwent [^11^C]PiB-PET, and positive amyloid-β accumulation was confirmed. Recently, a new research framework has been published in NIA-AA [23]; however, we did not apply it in this study because we did not have enough data on tau protein for the studied population. DLB was diagnosed using the fourth consensus report (the newest version) of the DLB Consortium [20].

Functional connectivity analysis can be used to assess the integration of brain activity across distant brain regions. The graph-theoretical method can provide functional connectivity using time-series spatially parcellated data in functional MRI. Our idea is to input standardized patient-series data in FDG-PET to generate metabolic connectivity. The advantage of the graph-theoretical method combined with the standardized data is that we can analyze the whole brain data simultaneously without operators’ controls. This analysis does not require predefined seeds or manual regions of interest. We can add the noble information of metabolism over conventional FDG-PET image. The correlation matrix and 3D schematic figures clearly exhibited the dense network in CN and the sparse network in AD and DLB, and the global measures were significantly decreased in AD and DLB compared with CN. Notably, the global measures were not significantly different, but patterns of sparsity of the correlation matrices and the 3D schematic figures were different between AD and DLB. The horizontal connections, the commissures on either side, were relatively preserved in DLB compared with AD; however, some of the connections were impaired more severely in DLB than in AD. These results suggest that DLB has restricted but deeper stereotyped network disruption than AD. We found several key nodes to differentiate DLB from AD, including the right posterior cingulate and the left transverse temporal gyrus. The right posterior cingulate was relatively preserved compared with the left posterior cingulate in early AD. Cerebral blood flow is left-side dominantly decreased in AD [24, 25]. The left hemisphere is language-dominant and is related to the progression of clinical symptoms in AD. Moreover, in DLB, glucose metabolism is preserved in the posterior cingulate (cingulate island sign) [6]. Because the other cerebral cortex has decreased metabolism, the connection between the posterior cingulate and the other linked cortex decreases, possibly leading to significant differences in the metabolic connection in the right posterior cingulate. The transverse temporal gyrus is left-dominantly associated with auditory processing and has a wide network to the white matter. A previous pathological study revealed that the left transverse temporal gyrus was relatively spared for alpha-synuclein deposit [26]. Accordingly, the connections between the left transverse temporal gyrus and the other regions potentially decrease. Interestingly, both the right posterior cingulate and the left transverse temporal gyrus are involved in important nodes of DMN. In earlier studies of functional connectivity using functional MRI, it is controversial whether the DMN connection in DLB is different from that in AD. Lowther et al. revealed that quite a few DMN connections were less in DLB than in AD [27]; however, Schumacher et al. found no decreased connection in DLB compared with AD [28]. Our study showed a part of DMN metabolic connections decreased more severely in DLB than in AD, suggesting a partially decreased pattern in the correlation matrix in DLB.

The metabolic connectivity also revealed that the left insula, left superior parietal, right transverse temporal, right superior temporal, and right entorhinal nodes had lower degrees in DLB than in AD. The insula had significant volume loss in prodromal DLB compared with CN [29]. The volume of the insula was preserved in AD. Superior parietal and temporal regions are key regions for visuospatial activities and construction of visual perception [30, 31]. The parietal region is involved in the key connection that mediates retrieval of object representation from long-term memory through visual imagery [30]. Visual processing deficit is one of the specific symptoms in discriminating DLB [20]. DLB patients with typical visual hallucination show reduced FDG metabolism in the right occipitotemporal cortex [32]. The entorhinal cortex plays an important role not only in the storage and retrieval for the episodic memories [33] but also in the visuospatial recognition [34]. Actually, the dysfunction of the entorhinal cortex could result in impaired visual recognition in patients with DLB [35]. Atrophy of the entorhinal cortex is more severe in DLB than in AD [36]. An earlier study investigating hippocampal subfield atrophy in DLB revealed thinning of the right entorhinal cortex [37]. Moreover, visuospatial attention functions in the right hemisphere dominance [38]. These nodes are supplementary nodes for differentiating DLB from AD.

This study has several limitations. A major limitation is a relatively small sample size, in particular in the DLB group. The small sample size might prevent us from detecting differences in topological parameters between DLB and AD, although the parameters could be significantly different. Second, the included patients in this study consisted of those diagnosed with AD or DLB only. Patients with combination-type dementia were strictly excluded from this study. The combination-type dementia of AD and DLB could show mixed-characteristic patterns of the topological model.
